# Outcomes for Implemented Macroeconomic Policy Responses and Multilateral Collaboration Strategies for Economic Recovery After a Crisis: A Rapid Scoping Review

**DOI:** 10.1177/00207314211007100

**Published:** 2021-04-05

**Authors:** Mark Embrett, Iwona A. Bielska, Derek R. Manis, Rhiannon Cooper, Gina Agarwal, Robert Nartowski, Emily Moore, Elena Lopatina, Aislinn Conway, Kathryn Clark

**Affiliations:** 1 3688 Dalhousie University, Halifax, Canada; 2 Nova Scotia Health Authority, Halifax, Canada; 3 3710 McMaster University, Hamilton, Canada; 4 Centre for Health Economics and Policy Analysis, 3710 McMaster University, Hamilton, ON, Canada; 5 School of Social Science, 1019 University of Aberdeen, Aberdeen, UK; 6 5620 McGill University, Montreal, Canada; 7 Cumming School of Medicine, 70401 University of Calgary, Calgary, Canada; 8 Better Outcomes & Registry Network (BORN) Ontario, Children’s Hospital of Eastern Ontario—Ottawa Children’s Treatment Centre, Ottawa, ON, Canada; 9 Children’s Hospital of Eastern Ontario Research Institute, Ottawa, ON, Canada; 10 12365 University of Ottawa, ON, Canada; 11 Michael G DeGroote School of Medicine, 3710 McMaster University, Hamilton, ON, Canada

**Keywords:** macroeconomics, multilateral coalition, economics, sustainable development, COVID-19, economic recovery

## Abstract

To promote postpandemic recovery, many countries have adopted economic packages that include fiscal, monetary, and financial policy measures; however, the effects of these policies may not be known for several years or more. There is an opportunity for decision makers to learn from past policies that facilitated recovery from other disease outbreaks, crises, and natural disasters that have had a devastating effect on economies around the world. To support the development of the United Nations Research Roadmap for COVID-19 Recovery, this review examined and synthesized peer-reviewed studies and gray literature that focused on macroeconomic policy responses and multilateral coalition strategies from past pandemics and crises to provide a map of the existing evidence. We conducted a systematic search of academic and gray literature databases. After screening, we found 22 records that were eligible for this review. The evidence found demonstrates that macroeconomic and multilateral coalition strategies have various impacts on a diverse set of countries and populations. Although the studies were heterogeneous in nature, most did find positive results for macroeconomic intervention policies that addressed investments to strengthen health and social protection systems, specifically cash and unconventional/nonstandard monetary measures, in-kind transfers, social security financing, and measures geared toward certain population groups.

## Introduction

In response to the COVID-19 pandemic, the United Nations (UN) has focused on gathering and dispersing knowledge in an effort to protect the population, limit lives lost, and build a better future. In March 2020, the UN released the UN Secretary-General’s “Shared Responsibility, Global Solidarity” report, which highlighted the severity of the global health crisis and the significant, predicted impact on economies worldwide.^1^ The UN called for an extensive and coordinated multilateral response equivalent to at least 10% of the global gross domestic product (GDP) to ensure the management of the crisis and reduce the risk of COVID-19 prohibiting economic recovery.^1^

Subsequently, in April, the UN released a report that described the immediate socioeconomic support that it would be providing globally in response to COVID-19. This framework included 5 integrated areas of focus, together aiming to protect and support individuals struggling as a result of the pandemic.^2^ The UN noted a specific desire to focus on countries, groups, and individuals most vulnerable to being overlooked. In this document, the UN stated that a significant portion of the funds dedicated to sustainable development goals (SDGs) and their associated sustainable development programs would be expanded and modified to meet needs corresponding to COVID-19, while remaining cognizant of the pledge of the 2030 Agenda (UN Framework for the Immediate Socio-Economic Response to COVID-19). These SDGs, adopted in 2015, act as a guide for the collaborative worldwide effort toward peace and prosperity. The 17 SDGs were created with the understanding that ending poverty will require strategies that also bolster health and education, reduce inequality, and promote economic growth, while simultaneously preserving biodiversity and addressing climate change (https://sdgs.un.org/goals). This article was commissioned in support of the development of a Research Roadmap for COVID-19 Recovery to accompany the aforementioned UN Framework for the Immediate Socio-Economic Response to COVID-19.^3^

Given the COVID-19 pandemic that has spread globally, governments around the world need to consider the implementation of significant macroeconomic policies to mitigate the negative effects and help stabilize their economies. The COVID-19 pandemic also accentuates the case for further development of international relations and collaboration for countries to support recovery.^4^ The world’s largest economies have adopted economic packages that include fiscal, monetary, and financial policy measures; however, the effects of these policies may not be known for several years or more. There is an opportunity to learn from policies made in the past to recover from other disease outbreaks, crises, and natural disasters that have had a devastating effect on economies around the world. This rapid scoping review aims to examine and synthesize peer-reviewed studies and gray literature that focus on macroeconomic policy responses and multilateral coalition strategies from past pandemics and crises to provide a map of the existing evidence for future policies and strategies.

## Objectives

This report supports the development of the UN Research Roadmap for COVID-19 Recovery,^3^ investigating Pillar #4 (“macroeconomic response and multilateral collaboration”) to

- identify and summarize existing research evidence relevant to the priority recovery areas;
- identify gaps in the available literature related to the equitable implementation of the recovery actions outlined in the report;
- describe the state of science and existing knowledge related to ensuring that the needs of the most vulnerable are met in the priority areas for recovery;
- describe the state of the science and existing knowledge of considerations of gender equity, racial equity, and environmental sustainability related to recovery;
- determine how available information and knowledge gaps differ across low- and middle-income and high-income countries.

## Research Question

What is the evidence, as well as gaps in evidence, assessing policy priorities and measures for implementing priority macroeconomic responses and multilateral collaboration to support COVID-19 pandemic recovery?

## Methods

We developed an a priori review protocol according to the research question. We drafted the protocol using the Preferred Reporting Items for Systematic Reviews and Meta-Analysis Protocols for Scoping Reviews (PRISMA-ScR),^5^ adapted by the research team, members of the Institute of Population and Public Health, and academic librarians with expertise in designing searches on topics relating to health and economics.

### Types of Study to Be Included

To be considered eligible for inclusion in the review, articles needed to include outcomes of implemented macroeconomic policies or multilateral coalition strategies. Macroeconomic policy strategies were identified as policies with outcomes related to financial system sustainability, fiscal, monetary, exchange policies, and social/environmental sustainability. Implementation of multilateral collaborative strategies had to focus on collaboration between 3 or more countries on the following types of strategies: customs procedures, monetary coordination, or environmental collaborative measures. Articles were also required to describe a macroeconomic or multilateral response to a disaster/crisis, including a financial crisis, environmental crisis, or natural disaster at a national, international, or global level. We included peer-reviewed journal articles if they were empirical studies, comparative studies, or scientific reviews and published between January 2015 and July 2020. We excluded non-English-language records. Quantitative, qualitative, and mixed-method studies were included to consider different policy outcomes. Gray literature was considered eligible if it met the same criteria as peer review, except for reviews and reports from international think tanks or consulting groups, which were also included. We excluded editorials, commentaries, letters, newspapers, and other types of opinion pieces.

### Search Strategy

#### Information Sources

To identify potentially relevant documents, the following databases were searched in July 2020: EBSCO: EconLit, ProQuest (ASSIA, Canadian Business & Current Affairs, Coronavirus Research Database), Business Premium Collection, Business Source Complete, and World-Wide Political Science abstracts.

A gray literature search plan was developed to incorporate 3 searching sources: (a) gray literature databases, (b) targeted websites, and (c) consultation with subject matter experts. The first search strategy involved searching gray literature databases relevant to the subject of the review. We searched: Econpapers, National Bureau of Economic Research, IDEAS, United Nations Digital Library, OECD databases, Center for Economic and Policy Research, Social Systems Evidence, and CADTH Gray Matters Health Economics guidelines. To identify additional relevant gray literature, the following websites were searched: International Labour Organization (www.ilo.org), World Bank (www.worldbank.org), Center for International Governance Innovation (www.cigionline.org), Center for Multilateralism Studies (www.https://www.rsis.edu.sg/research/cms), and the Commonwealth Library (https://www.thecommonwealth-ilibrary.org/). Because databases can use variable search functionality and filters to retrieve results, the researchers adapted search terms. An example of the search strategy can be found in Supplemental Appendix 1. Modifications and truncations were used to fit the formatting requirements of specific search engines. Terms related to macroeconomic recovery or multilateralism were combined with terms related to crises or disasters.

#### Selection of Sources of Evidence

The found articles and gray literature were divided among team members for the title and abstract screening, using the stated eligibility criteria. Team meetings were held to discuss any questions or concerns regarding eligibility. We completed the screening using Microsoft Excel. After title and abstract screening was completed and the results were compiled, full-text screening commenced on the potentially eligible articles. Each member screened the full-text articles using the same eligibility criteria applied to the full text. Online team meetings were held to discuss any uncertainties regarding eligibility.

### Data Extraction

We proceeded with data extraction from full-text articles that were deemed eligible for inclusion. Team members were assigned specific articles to extract. Team meetings were held to discuss any questions or concerns that arose during extraction. We extracted data from eligible studies using an a priori-designed data extraction sheet in Microsoft Excel. The team members received a legend describing each data point for reference.

The following data and key findings were extracted.

#### Study Characteristics

- *General information:* title, authors, journal, database, digital object identifier, and year.
- Study characteristics (eg, design, data collection methods).
- *Intervention/strategy characteristics:* macroeconomic/multilateral coalition intervention types.
- *Location of study:* country, city, region, and global.
- *Outcomes:* fiscal policies (changes in the level and composition of government spending, the level and types of taxes levied, capital expenditure, spending, taxes and transfers on private consumption, investment, and net exports), monetary policy (cash rate), exchange rate policy (value of the domestic currency).
- *Strategies:* description and implementation approaches used in responses.

#### Macroeconomic Policy/Intervention Categories

- Investments to strengthen health and social protection systems and move toward universal health care and universal social protection systems.
- Spending and investment policies (tax deferrals, postponing social contribution payments, wage subsidies, temporary suspension of loan repayments, loan guarantees, subsidized loans, or direct grants).
- Financial system stabilization policies (banking, central bank coordination, interest rates, payment deferrals).
- Fiscal and debt policies toward economies (debt relief, deferral of debt payments, tools of debt sustainability, debt restructuring mechanisms).
- Social and environmental sustainability: policies to reduce carbon emissions and reduce waste and policies that address social values and community engagements.

#### Multilateral Coalition Strategy Categories

- Cooperation on trade policy: coordination and harmonization, impact on supply chains.
- Customs procedures, including importing/exporting policies and agreements.
- Monetary coordination, involving central banks of countries working together.
- Macroeconomic objectives, including inflation, consumption, growth, and liquidity.
- Environmental collaborative measures: policies related to environmental protection measures.

## Results

After duplicates were removed, we identified 799 citations from searches of electronic databases and reference checking. We identified 558 gray literature records. Based on the title and abstract screening, we excluded 1212 items with 145 full-text articles being retrieved and assessed for eligibility. Of these, we excluded 119 records. We excluded 2 additional articles because we were unable to retrieve the full-text version. The remaining 24 records were considered eligible for this review. The adapted PRISMA flow diagram is shown in Supplemental Appendix 2. The characteristics of the included sources are presented in Table 1.

**Table 1.** Characteristics of included sources (*n* = 23)

Characteristic	*n* (%)
Study design	
Empirical study	8 (35)
Comparative study	4 (17)
Other	11 (43)
Year	
2010	4 (17)
2011	1 (4)
2012	1 (4)
2013	2 (9)
2014	2 (9)
2015	3 (13)
2017	1 (4)
2018	4 (14)
2019	2 (9)
2020	3 (13)
Country classification	
High income	9 (39)
Low/middle income	14 (61)
Crisis	
Financial crisis (2008)	17 (74)
COVID-19 (2020)	2 (9)
Other	4 (17)
Macroeconomic policy domain (some papers deal with more than one)	
Social and environmental sustainability	2 (9)
Financial system stabilizing	6 (26)
Fiscal and debt	2 (9)
Investments to strengthen health and social protection	4 (14)
Spending and investment	6 (26)
Other	1 (4)
Multilateral coalitions domain (some papers deal with more than one)	
Monetary coordination policies	2 (9)
Trade cooperation	1 (4)
Environmental collaborative measures	0 (0)
Other multilateral coalition strategies	1 (4)

## Macroeconomic Policies and Outcomes

We included 22 studies and gray literature reports that described macroeconomic policies and outcomes. The findings from these studies and reports were classified according to the following categories: (a) investments to strengthen health and social protection systems, (b) spending and investment policies, (c) financial system stabilization policies, (d) fiscal and debt policies, (e) social and environmental sustainability, and (f) other macroeconomic policies.

### Investments to Strengthen Health and Social Protection Systems

Four international reports examined the impact of macroeconomic investments made to strengthen health and social protection systems. The majority of the documents described the actions taken following the financial crisis of 2007 to 2008. Measures taken in response to other crises, such as the human immunodeficiency virus (HIV)/acquired immunodeficiency syndrome (AIDS) epidemic and the Ebola virus disease outbreak, were also mentioned.

Tirivayi et al^6^ rapid review prepared as part of the United Nations International Children's Emergency Fund (UNICEF) Office of Research was written to synthesize evidence on the effects of past policy responses to multiple crises concerning children and families and to make recommendations for policy interventions during the COVID-19 pandemic. Overall, their rapid review found that the effects of social protection interventions following crises have not been assessed uniformly across various topics and jurisdictions, with most of the available articles focusing on the impacts of social assistance programs on children and families. The available evidence of economic outcomes and social protection responses is not the same across regions. The rapid review provided key findings that could be implemented in response to the COVID-19 pandemic. These included ensuring that social protection and economic stimulus policy responses took into account the well-being of children and addressed gender inequality; multigenerational impact was considered; existing infrastructure and programs were expanded along with the introduction of new programs; policy responses took into account at-risk and informal workers and their families; health care access was maintained, and the possible negative effects of austerity policies on children were anticipated.

Global case studies of interventions were presented in the rapid review.^6^ Among these were measures taken in the context of the HIV/AIDS pandemic, particularly in Sub-Saharan Africa, where in-kind transfers (eg, food aid), cash transfers, and support for caregivers of orphaned children were implemented. The interventions were effective at improving the conditions for children and families, chiefly, their living circumstances, food security, health care access, and education. The effects of similar social protection measures were addressed in the context of another health crisis: the Ebola virus disease outbreak in Sierra Leone. It was noted that these interventions, such as including cash transfers, in-kind transfers, educational support, and/or jobs, had positive effects on disease survivors, 2 years later, especially on food security and emotional well-being. Furthermore, interventions that had greater intensity and duration were found to result in more significant effects.

In the same report, the impact of investments in health and social protection systems made in response to the financial crisis of 2007-2008 was researched in detail. The crisis led to social protection and expansionary fiscal responses in the short term, followed by austerity measures in the long term. Among the interventions introduced following the financial crisis, the International Labour Organization and the International Institute of Labour Studies report mentioned the case of Indonesia, where infrastructure stimulus efforts of US$157 billion mostly focused on public works infrastructure.^6^ These efforts resulted in employment opportunities, particularly in rural areas and among disadvantaged groups. Australia’s response to the financial crisis, which included 3 cash transfers for working individuals and families, resulted in greater equality for low- and middle-income families and reduced poverty levels following the crisis.^6^

Martorano’s^7^ report on “The Consequences of the Recent Economic Crisis and Government Reactions for Children,” prepared as part of the UNICEF Office of Research, further analyzed the impact of various European government policies on poverty reduction and income distribution in response to the economic crisis, focusing on children. Many European governments reacted similarly to the macroeconomic shock resulting from the crisis. In-cash benefits, the provision of one-off payments to low-income families, the reformation of school and childcare benefits, as well as parental leave policies, resulted in a sharp increase in public spending and kept equality stable. Generally, European countries initially resorted to stimulus packages but when these failed, austerity measures were taken that resulted in decreased public expenditure spending. Austerity measures (ie, increasing indirect taxation, increasing direct taxation, reduction of family benefits, reduction of social allowances) tended to burden the most vulnerable groups, as they resulted in a decline in living conditions and cuts in funding for social services, education, and health.

As part of the International Labour Office’s report, Bonnet et al^8^ examined the social protection policies implemented by 77 countries postfinancial crisis, including social protection schemes (contributory and noncontributory), cash and in-kind transfers, social security financing changes, targeted measures geared toward certain groups (migrant, irregular, temporary workers), and minimum wage regulations. Social protection measures represented long-term strategies postcrisis, with short-term strategies geared toward adjusting existing schemes. Investing in social protection schemes mitigated the adverse effects on unemployment and poverty levels, despite the associated costs. Cash transfer programs (conditional and unconditional) were the most prevalent postcrisis and were shown to be effective at reducing poverty.

Moreover, the report examined interventions classified according to 5 categories: unemployment benefits, pensions, health care, social assistance, and other programs (minimum wage, migrant worker assistance, child and family benefits, housing benefits).^8^ Examples of policy outcomes (ie, reduced unemployment) were presented for a subset of the 43 countries examined in detail. Countries with unemployment insurance systems tended to expand these systems to replace the income for unemployed individuals; numerous European countries expanded partial unemployment benefits (eg, reduced hours); middle-income countries tended to expand public employment and cash transfer schemes; low-income countries tended to expand food subsidies (eg, food cards). One or more expansionary policies resulting in fiscal consolidation to counter public debt were implemented in 41 of the 43 countries examined in detail. A total of 25 countries indicated lower contributions to social security.

Wagner et al^9^ conducted a comparative study measuring the impact of the President’s Emergency Plan for AIDS Relief (PEPFAR) on employment throughout 10 countries in Sub-Saharan Africa, as well as the population-level economic effects of antiretroviral therapy. Over a period of 10 years, US$54 billion in support was authorized by the American Congress. The authors found that HIV-related mortality in countries receiving aid was decreased comparing to HIV-related mortality in control countries. Increased employment levels were also noted, particularly among males. Furthermore, PEPFAR resulted in substantial economic payoff with 9 percentage points increases in male employment levels per US$100 increase in funding per person.

### Spending and Investment Policies

In terms of analyzing spending and investment policies implemented in response to crises, 6 articles were identified, the majority of which dealt with the effects of policies implemented during the financial crisis of 2008.

Islam and Verick^10^ reviewed the causes, impacts, and policy outcomes of the financial crisis across the globe. The authors identified 3 categories of interventions: (a) credit injections of money and bailouts, (b) interest rate cuts, and (c) increased fiscal spending. Two case studies of G20 countries were presented to demonstrate the effects of policies. The fiscal stimulus package in Indonesia centered around the reduction of income and indirect taxes, investments in infrastructure, and subsidies. It resulted in 1 million jobs. Next, the American Recovery and Reinvestment Act involved tax reductions, government purchases, and transfers. It led to increased employment levels, although the authors noted that the outcomes were modest.

The impacts of the American government's stimulus package following the 2008 financial crisis were examined in greater detail in 2 reports: “The Economic Impact of the American Recovery and Reinvestment Act—Five Years Later.”^11^ and “The Economic Impact of the American Recovery and Reinvestment Act of 2009.”^12^ The reports overviewed the implemented macroeconomic interventions, including investments in employment opportunities, emergency assistance, technology, science, health, transportation systems, infrastructure projects, and environmental protection, as well as the stabilization of local and state budgets. The reports concluded that the American Recovery and Reinvestment Act of 2009 was able to move the economy into a much more favorable position over the 4 years following the crisis through the formation of “6.4 million additional job-years,” annual decreases in unemployment rates of 0.8 percentage points, and increases in GDP. The reports noted that even though certain measures were temporary, such as cuts to the payroll tax, businesses were able to rebound postcrisis due to their implementation.

It is also important to note that “The Economic Impact of the American Recovery and Reinvestment Act of 2009” described domestic government macroeconomic policies that could have had worldwide impacts. Countercyclical fiscal policy could benefit all trading partners as well. For example, the G20 international stimulus packages of 2007- 2009 benefited the U.S. economy, and other countries saw benefits of the Recovery Act. For example, in 2009, the U.S. GDP raised 1.9%, by ∼1.5% being attributed to domestic policies and 0.4% being a result of fiscal stimulus in emerging Asian countries (0.3%), Japan (0.1%), Europe (0.1%), and other countries (0.2%). Additionally, U.S. domestic stimulus policies were estimated to have raised GDP globally by 0.7% in 2009 and 2010.

Measures aimed at maintaining employment levels were further reviewed by Clemens,^13^ who examined labor market institutions and levels of young adult employment during the 2008 financial crisis among 23 countries classified as high income, long industrialized. The analysis showed that institutional factors affected macroeconomic conditions and the market's response to them. Clemens found that wage floors set by legislation remained constant during the crisis and, as a result, their levels were not affected by decreases in demand. The employment levels of young adults did not decrease as much in countries where wage floors were determined via collective bargaining agreements in comparison to those set by legislation.

Employment maintenance measures were overviewed in the International Labour Organization and the International Institute of Labour Studies report.^14^ Germany’s Kurzarbeit program allowed workers to maintain their jobs with reduced hours with the benefits and costs of the program distributed among the workers, their employers, and the government. The report also found that among countries that performed successfully following the economic crisis, a combination of fiscal policies, programs aimed at protecting employment, and good levels of social dialogue resulted in job maintenance and creation and allowed jurisdictions to meet their financial goals. Government investments in programs aimed at stimulating the labor market also resulted in further employment opportunities and increased labor incomes.

Last, the effects of active labor market policies, which assist individuals with remaining in the labor market or incentivize the seeking of employment opportunities, were reviewed in a policy brief prepared by the International Labour Organization to inform actions during the COVID-19 pandemic.^15^ The policy brief noted that these interventions were particularly effective in high-income countries during recessions, whereas in Latin America and the Caribbean countries, they were more effective in times of economic expansion due to the resources involved. Based on the previous literature, it was determined that successful programs implemented across various jurisdictions lasted for at least 4 months, were targeted at certain groups, and responded to labor market needs locally. The report concluded that based on past evidence globally, active labor market policies could be implemented throughout the COVID-19 pandemic to support employment levels.

### Financial System Stabilization Policies

Six articles described financial system stabilization policies after a crisis or disaster. Lenza et al^16^ described the response of 3 central banks—the European Central Bank, Federal Reserve, and Bank of England—to the 2008 financial crisis from 2007 to 2009. They focused on the banks’ nonstandard monetary policy measures that allowed them to provide a large volume of liquidity to a sufficient number of counterparties so they could settle transactions across the central bank balance sheet. A common feature of nonstandard measures to which they referred as quantitative easing, which allows central banks to expand their balance sheet (and in turn, their monetary base), while not altering their asset side of the balance sheet. A difficulty in assessing the nonstandard measures is that “the nonstandard measures employed by the 3 central banks are no longer easily characterized as either one,” thereby making it difficult to distinguish the impact of individual measures. Central banks also conducted standard measures by offering facilities for other banks to refinance liquid assets, thereby avoiding a “fire sale” of these liquid assets that would have further eroded the banks’ capital and destabilized the markets. In October 2008, the 3 central banks, along with others, also implemented a 50-basis point cut in their key policy rates (a standardized measure). The results of these policies caused interest rates to fall and helped manage the banks’ balance sheets. Resultantly, central bank intermediation using nonstandard measures has played a quantitatively significant role in stabilizing the financial sector and economy after the 2008 financial crisis. Over time, this action succeeded in narrowing the spread between the unsecured interbank deposit rate and the overnight interest swap rate (a proxy for secured rates). It resulted in a widening of the spread of the overnight money market rate below the conventional policy rate and helped to flatten the money market yield curve.

Satiroglu et al^17^ explored the covered interest rate parity (CIRP) condition to determine how the Federal Reserve and U.S. Treasury’s economic policies affected global liquidity at the time of the 2008 financial crisis. They reviewed federal swap lines intended for developed and emerging markets through bilateral currency arrangements with the intent to reduce stresses associated with dollar funding abroad. The authors determined that for short-term maturities, swap line extensions decreased CIRP deviations in emerging markets. In developed markets, this was the case for short- and long-term maturities. These policy measures were effective at limiting funding liquidity constraints. The CIRP condition was significantly restored through swap line extensions. The authors noted that their findings indicated that “swap facilities are an important tool for minimizing systemic liquidity disruptions and are also effective in restoring the link between price and fundamentals for both developed and emerging markets.” Furthermore, the authors stated that several of the reviewed programs “tried to compensate for capital losses sustained by U.S. banks,” while also improving the state of funding in developed market economies. Overall, the U.S. Treasury's programs affected secured markets in comparison to the Federal Reserve's programs, which affected unsecured markets.

Abildgren et al^18^ investigated the impact of significant events—for example, the European sovereign debt crisis, the 2008 financial crisis, and negative interest rates—on Denmark’s uncollateralized interbank market. The primary response was the central bank's decision to move its interest rate to below zero. This affected trading, interconnectedness, interest rates, and the financial distress on banks. There was a decrease in trading activity since the 2008 financial crisis, and this policy helped increase the number of large transactions. During the crisis, there were fewer banks as a result of acquisitions, mergers, and failures. Larger institutions did well; however, small- and medium-sized banks only had 5% of the lending market, and many failed. Denmark's largest banks became systemically important financial institutions. From month to month, these top institutions for borrowing and lending would change, suggesting that they centralized the market as a group rather than individually. On uncollateralized day-to-day loan contracts, the “spreads between the highest and lowest overnight interest rates” showed that “risk premiums were high during the peak of the crisis in 2008.” Among banks, interest rates did not differ greatly, which indicated that they may have assessed and valued “credit and liquidity risks” using similar methods. Banks paying higher interest rates were more likely to get into distress. When comparisons were made by other banks, it was possible to identify signs before the banks made an official statement. Overall, this approach led to fewer small- to medium-sized banks. It was noted that monitoring the spread of interest rates on uncollateralized day-to-day loans could have helped identify banks in distress for up to 2 years before they made it public.

Benediktsdóttir et al^19^ described the Icelandic banking system to explore the policy decisions that led to its recovery from the 2008 financial crisis. The emergency legislation enacted following the crash gave depositors priority and allowed the Icelandic Financial Supervisory Authority to intervene in the operations of the banks, which ultimately resulted in its takeover of the country's 3 largest banks. The controls placed restrictions on foreign investments and disallowed foreigners with assets in krónur from their recovery, thereby preventing capital outflows and the collapse of the national currency. Iceland used an effective multicomponent strategy of legislative activities, capital controls, alleviation of risks, and maintenance of financial stability to successfully recover from the financial crisis. By 2017, Iceland had reclaimed its position as “the fourth-richest country in the world in terms of GDP per capita,” has dropped to 21st place in 2010. One point that the authors make was that given Iceland's relatively simple economy and own currency, their findings may not have been transferable to other settings.

Chen et al^20^ overviewed the actions taken globally following the 2008 financial crisis, both immediately and in the medium term (2015-2017). The authors found that the combined use of unconventional monetary policies (asset purchases, liquidity supports) through the fiscal stimulus packages of central banks supported economic growth. They also found that quantitative easing (the purchasing of government bonds, corporate bonds, and mortgage-backed securities to expand balance sheets), in addition to rate hikes when unemployment and inflation hit a specific level, negative interest rates on excess reserves, and yield curve control, had varying effects on financial conditions and interest rates. Generally, the authors found that the policies had a positive effect on domestic output and trading imports. Quantitative easing, they considered, helped stabilize markets by reducing the risk of declines in asset price. Although these measures were viewed as positives for developed economies, there was controversy regarding their effectiveness in emerging and developing economies.

The International Monetary Fund policy article (2013) reviewed unconventional monetary policies, including policies aimed at restoring the market and supporting economic growth, following the financial crisis of 2008.^21^ It was found that in countries that adopted these measures, positive effects were seen on inflation and economic activity. Furthermore, the measures were also beneficial globally. Nonetheless, it was noted that unconventional monetary policies had associated risks, including the credibility of central banks, financial stability, and reform agency complacency, thereby necessitating structural reforms to sustain growth.

### Fiscal and Debt Policies

Three reports overviewed the effectiveness of fiscal and debt policies following the financial crisis of 2008. MacMillan^22^ from the Commonwealth Secretariat reviewed the macroeconomic responses after the crisis. They analyzed government spending measures targeted at increased investment or consumption, transfers, and targeted tax cuts and found that these policies were more effective in comparison to general subsidies or tax cuts. Furthermore, the authors found that in developing countries, measures aimed at increasing expenditure should be emphasized in comparison to tax rate reductions, due to limited revenue ratios. Finally, they concluded that in general, investments in fiscal policies (specifically infrastructure) have helped governments respond, while stimulus packages worked for an economic rebound.

Liang et al^23^ provided an overview of the fiscal policy actions taken by the American government in response to the financial crisis of 2008 in their discussion report. Specifically, they reviewed fiscal and monetary interventions aimed at supporting credit and growth, including cutting interest rates from 5.25% to 3.00% and implementing the Economic Stimulus Act, which consisted of US$100 billion in household tax rebate checks. They found that from 2007 to 2009, GDP decreased by 4% and unemployment levels increased from 4% to 10%. GDP recovered 6 years later and by 2018, it was 15% higher than before the crisis. The conclusion was that the American government's fiscal policy actions during the crisis were effective at preventing “a second Great Depression.”

The World Bank Group studied the effects and credit market impact of India’s Agricultural Debt Waiver and Debt Relief Scheme, a bailout program geared toward the rural sector in response to the 2008 financial crisis.^24^ The main policy focus was on borrowers who were in default on their loans, with debt relief eligibility based on the size of landholding. The bailout program had a significant negative effect on the repayment of loans among those individuals who did not qualify for the program, perhaps because they were anticipating another round of bailouts. Although the program was expected to address debt overhang and strengthen household balance sheets, there were no improvements in agricultural productivity, rural wages, or household consumption. Instead, the result of the bailout was a reallocation of credit and greater defaults. Following the program, loan performance worsened in districts with greater program exposure, and this was not found to be driven by the bank sector. Finally, postprogram loan defaults appeared to respond to the electoral cycle.

### Social and Environmental Sustainability

Two studies investigated environmental or social sustainability policies. Klyviene and Kedaitiene^25^ examined the effects of environmental policies on changes in GDP, employment, investment, and trade in 19 Eurozone countries between 2000 and 2016, which included the period of the financial crisis. The authors found that environmental policies designed to decrease the per capita CO_2_ emissions in Eurozone countries negatively affected the GDP in the short term, but over a longer period, the effect was positive. During the financial crisis, austerity policies aimed at energy savings were put into place and GDP was noted to grow between 2005 and 2016. The authors also found evidence that environmental policies may be beneficial for international trade. Unable to make conclusive statements regarding the impact of environmental policies on employment and investment, the authors called for further research to explore these areas.

Secondly, the Environmental Protection Agency in the United States administered programs funded by the American Recovery and Reinvestment Act of 2009, which was implemented following the 2008 financial crisis. The programs included the cleanup of land contamination, reductions in air pollution, and increases in water quality through infrastructure projects aimed at decontaminating drinking and surface water.

### Other Macroeconomic Interventions

One report examined other macroeconomic interventions arising from the financial crisis of 2007-2008.^26^ It examined the recovery from the crisis among a selection of Organization for Economic Co-operation and Development countries, focusing on regulatory reform, market openness measures, and competition policy. For example, in South Korea, the Temporary Regulatory Relief program delayed the inception of 280 regulations for 2 years to promote investments from the private sector and to decrease the burden on businesses. Citizens and businesses reported high satisfaction with the outcome of the policy. The conclusions from the report were that regulatory reform during a crisis was often effective in stimulating the economy.

## Multilateral Coalition Policies and Outcomes

Four articles identified in this review discussed multilateral coalition policies. We also decided to include 1 additional record, a plurilateral agreement, because it focused on more than 3 countries within a multilateral entity. Out of those, 2 articles discussed monetary coordination policies, and 1 article discussed trade cooperation policies, policies focused on avoiding market failure through international collaboration, and one was classified as “other multilateral.”

### Monetary Coordination Policies

Currency swap lines established by the U.S. government with the European Central Bank and the national banks of other countries in response to the economic crisis (2007-2009) were discussed in 2 articles.

Satiroglu et al^17^ also evaluated the extension of currency swap lines to international central banks in both developed and emerging markets through bilateral currency arrangements to alleviate dollar funding stresses internationally. Through swap lines, banks in need of U.S. dollars received discount window funding that significantly reduced CIRP deviations, but only for short-term maturities. While the extension of swap lines to developed markets significantly reduced CIRP deviations for both short- and long-term maturities, the authors concluded that swap lines represented a utilitarian tool in addressing CIRP violations and, thus, relieving foreign exchange market frictions.

Sahasrabuddhe^27^ examined the decision-making process of the U.S. Federal Reserve in selecting emerging market swap line recipients to receive liquidity injections in response to the 2008 financial crisis through bilateral arrangements known as Central Bank Liquidity Swaps. The strategic motivations underlying the U.S. Federal Reserve’s decision were explored, and Sahasrabuddhe argued that the U.S. Federal Reserve was more likely to grant a swap to economies that shared its policy preferences for greater capital account openness. The author also posited that the relationship between swap recipients and shared policy preferences was mediated by political and diplomatic considerations. The article concluded that the U.S. Federal Reserve strategically chose its emerging economy partners to reinforce economic alliances, focused particularly on those who experienced an increased influence in economic governance after 2008. Outcomes of this monetary coordination were not discussed.

### Trade Cooperation

The report by Dengler and Hoekman^28^ investigated the association between involvement in international trade agreements, the World Trade Organization’s (WHO) Government Procurement Agreement (GPA), and several preferential trade agreements (PTAs) and public-sector imports after the 2008 financial crisis. It examined the impact of GPA membership on public import shares and sustaining public-sector openness. The authors did not investigate the impact of GPA membership in the private sector. Countries that were members of the GPA reported higher import shares after the crisis. The authors noted that GPA may have prevented protectionism when the economy was suffering and encouraged knowledge sharing. The GPA supported members in the delivery of public-sector openness, and the authors found that public procurement provision PTAs and the GPA could act as substitutes in the process of making this more sustainable. Although many countries were members of the GPA at the time of the study, developing countries were underrepresented.

### Other Multilateral Coalition Strategies

Kim^29^ analyzed the WHO response to the HIV/AIDS epidemic. Following an initial period of global inaction to address HIV/AIDS, the WHO developed norms in the early stages of the epidemic to guide the worldwide response to HIV/AIDS. A coalition led by the WHO developed a framework for an HIV/AIDS response that featured 4 components: (a) sharing information and enhancing awareness, (b) constructing perception and cooperation, (c) establishing norms in the UN system, and (d) mobilizing and strategizing to implement norms. In addition to having a positive effect on the population affected by the epidemic, this approach activated and boosted the provision of financial support from major donors across many countries.

## Inclusion of Vulnerable Populations and Sustainable Development Goals

Five articles discussed vulnerable populations.^3–5,11,27^ Of these, 2 focused on macroeconomic policies in a global context, while 4 articles discussed one or more countries. Four of the studies included a mixture of low-, middle-, and high-income countries, and 1 examined only high-income countries. While all 5 articles made mention of vulnerable populations that may have been impacted during a crisis, 3 of the articles specifically centered their studies around vulnerable populations.

Three distinct groups of vulnerable populations were mentioned: children/youth,^3–5^ emerging/developing countries,^3–5^ and low-income groups.^11,27^ Statutory social assistance programs were identified as being able to cushion children from the negative economic impacts of past economic crises.^3^ A similar finding was described in a study conducted by UNICEF in 2014 with a focus on the past financial crisis in Europe.^4^ The provision of one-off payments to low-income families was discussed as a spending and investment policy that could have had positive impacts on children. Relatedly, the International Labour Office (2011) studied social protection policies that were implemented by 77 countries postfinancial crisis.^11^ Specifically, the report focused on how unemployed youth were disproportionately impacted postfinancial crisis. For example, it noted that Argentina extended unemployment benefit coverage to youth and implemented a noncontributory social protection system for children and youth living in unemployed homes.

One article mentioned the SDGs.^22^ The empirical study aimed to investigate the possible effect of environmental policies on macroeconomic variables such as GDP, investment, employment, and trade.^22^ Overall, the research group concluded that environmental and socially sustainable policies led to a short-term negative effect on GDP, although the long-run economic effects could have been positive ones.

## Inclusion of Equity-Related Concerns

The previously described report by Tirivayi et al examined government actions in response to the 2008 financial crisis and analyzed the impacts of various policies on income distribution and poverty within Europe. Most governments in Europe enacted similar policies by initially implementing blanket stimulus packages, followed by austerity measures when the stimulus packages failed to rescue the economy.^4^ The most common stimulus measures were changes to cash benefits paid, one-time payments to low-income families, and reforming school and childcare benefits. Common austerity measures included an increase in direct or indirect taxes universally, as well as cuts to education, social funding (ie, social protection expenditure, housing, unemployment benefits), and health spending. Also, it was found that the switch from stimulus to consolidation policy created inequitable redistributive consequences that served to widen the inequality, creating a decline in living conditions for individuals considered to be in vulnerable groups.

Furthermore, the authors concluded that the response differed across regions, outcomes, and types of policies. Many countries across different income levels implemented one-time, in-kind payments targeted to a variety of populations, including low- to middle-income families, to support access to education, food, and employment. They found that cash transfers provided important benefits, such as well-being and access to food. Cash transfers to low- to middle-income families were preferred to a tax benefit system as cash transfers were better at targeting low- to middle-income families.^3^ In Sub-Saharan Africa, in-kind cash payments during the crisis alleviated negative consequences related to HIV illness and improved access to education, health, childhood development, and household food security. Indonesia established social health insurance during the financial crisis, which improved access to both inpatient and outpatient health care. This access, however, was greater in the contributory group (ie, public and private wage employees, informal workers, and nonworkers) compared to the noncontributory group (ie, poor families, the disabled). The authors concluded that investments that strengthen health and social protection are cash transfers targeted to vulnerable populations, and these transfers can lessen the magnitude of crises by reducing poverty and food insecurity and protecting health.

As mentioned earlier, Dengler and Hoekman^28^ analyzed the effect of GPAs and PTAs and reported that these memberships led to member countries having a higher import share following the 2008 financial crisis. There were significant equitable differences in the import shares between member and nonmember countries, but not between member countries. As discussed in the report by Kim^29^, the WHO, in raising and coordinating funds as well as developing norms, played a central role in ensuring equitable access to funds and resources amid the ongoing HIV/AIDS crisis.

## Discussion and Funding

### Summary of Evidence Found

The evidence reviewed in this rapid review demonstrates that macroeconomic and multilateral coalition strategies, when implemented, have various impacts on a diverse set of countries and populations. As the included articles and reports present heterogeneous data, they cannot be synthesized together to draw firm conclusions. However, most of the presented evidence indicated positive results from macroeconomic intervention policies that were implemented following various crises to strengthen health and social protection systems, specifically, cash and unconventional/nonstandard monetary measures, in-kind transfers, social security financing, and measures geared toward certain population groups. The majority of the included articles focused on macroeconomic policy interventions implemented after a crisis/disaster, rather than multicoalition strategies, although some considered both. The most prevalent interventions were cash transfer programs and unconventional/nonstandard policies (conditional and unconditional).

In terms of the settings of the interventions, 9 articles addressed policies implemented in a mix of high-income countries. Fourteen reports focused on lower- or middle-income countries or a mix of both settings. Studies on lower-income countries focused on investment in health and social protection (4), spending and investment policies (1), and fiscal and debt policies (1).

### Summary of Evidence Gaps

Several topics were not addressed in the included articles. Only 2 articles addressed macroeconomic policy interventions that examined environmental and social sustainability. In addition, just 2 of the included articles discussed fiscal and debt policy. For the category of “other macroeconomic interventions,” we found 1 publication that focused on regulation.

There was an overall lack of studies on multilateral coalition (4 articles in total). Monetary coordination was addressed most often (2 articles), while only 1 article was found that focused on environmental collaborative measures, trade cooperation, or other multilateral coalitions. Only 2 of these articles were empirical studies, and the remaining were policy reviews. We did not find any articles on environmental collaborative measures. This indicates a gap in empirical evidence regarding multilateral coalition strategies.

Only 5 articles addressed equity issues, which were not the main focus of the reports. One article covered SDGs. Aside from the article by Tirivayi et al, which recommended social protection and stimulus package responses addressing gender inequality, no other article addressed racial or gender equity. This signals a significant gap in both macroeconomic and multilateral coalition published research that addresses gender or racial issues. Furthermore, the inclusion of just 1 study that focused on low- and middle-income countries indicates a gap in analyzing the effects of macroeconomic policies in these settings.

Furthermore, there was a lack of systematic reviews and other forms of scientific reviews. Although the quality of evidence was not assessed, the study designs of the included articles were categorized; quality of evidence can be assessed based on study design with network meta-analysis, systematic reviews, and randomized control trials being top quality and observational studies and case studies being lower quality.^30^ Although 1 network meta-analysis was included, no other type of scientific review was found. The remaining articles could be assessed as observational, comparative, or nonscientific review (gray literature). Recognizing that economic study designs may often be observational, more systematic reviews and network meta-analyses would benefit the field. Furthermore, no causal studies were found, only correlational studies related to policies and their outcomes. Many studies also did not provide an in-depth description of policies or their outcomes on recovery. This may be due to the fact that no government policy or strategy works in a vacuum; instead, they are affected by existing policy and future policies.

### Limitations

There are several limitations to this study, including the lack of quality of evidence or risk of bias assessment. Furthermore, given the narrow timeframe to complete the rapid review and the single screening and extraction processes, there is a possibility of errors at both stages, in which relevant articles may have been improperly excluded or information may have been overlooked. Despite these limitations, the strength of the search strategy and the number of databases and gray literature sites searched may help mitigate this risk. In terms of the studies themselves, many of the articles that included data from multiple countries were not systematic or transparent about case selection or data collection, which may heighten the risk of bias.

### Conclusion

There are no doubts that the severity of the current global COVID-19 health crisis will have a significant impact on economies worldwide, and thus on the people that rely on the economy for work, assistance, and welfare support. Although the results of this review provide the UN roadmap with existing evidence of the impact of macroeconomic policies and multilateral coalition strategies, it also revealed evidence gaps that underscore the importance of improving the examination of COVID-19 recovery activities and policies. The scope and scale of macroeconomic policies implemented during the COVID-19 pandemic need to be carefully assessed to better understand the equity of their impact, specifically on high-risk and marginalized populations. Long-term impacts and implications of the trade-offs of policies (ie, eventual austerity measures) will also be critical to avoid future economic and social instability.

## Supplementary Material

Supplementary material
