# Supplementary material for: Outcomes for Implemented Macroeconomic Policy Responses and Multilateral Collaboration Strategies for Economic Recovery After a Crisis: A Rapid Scoping Review
Source: Int J Health Serv. 2021 Apr 5;51(3):337–49. doi: 10.1177/00207314211007100 (PMC8193076; doi:10.1177/00207314211007100)

Appendix 1: Sample Search Strategy

**Search terms:**

“macroeconomic” OR “macroeconomic recovery” OR “fiscal recovery” OR “economic impact” OR “macroeconomic policy” OR “fiscal policy” OR “debt relief” OR “monetary measures” OR “monetary policy” OR “coronanomics” OR Macrodynamics OR Macroeconomics OR Macroeconomy[1] OR “multilateral coalition” OR “International coordination “ OR “regional cooperation” OR Globalization OR “trade policy” OR “international collaboration” OR “multilateral collaboration”

AND

“Coronavirus” OR “COVID-19” OR “SARS-CoV-2” OR “2019 novel coronavirus” OR “2019-nCoV” OR “Wuhan coronavirus” OR “novel coronavirus” OR “Wuhan virus[2] ” OR pandemic OR disasters OR pandemics OR epidemics OR “disease outbreaks” OR ebola OR zika OR sars OR mers OR HIV OR AIDS OR “subprime mortgage crisis” OR “2008 financial crisis”

In gray literature databases, some modifications were used if the search string was too long for the search engine.

Appendix 2: Adapted PRISMA Flow Diagram


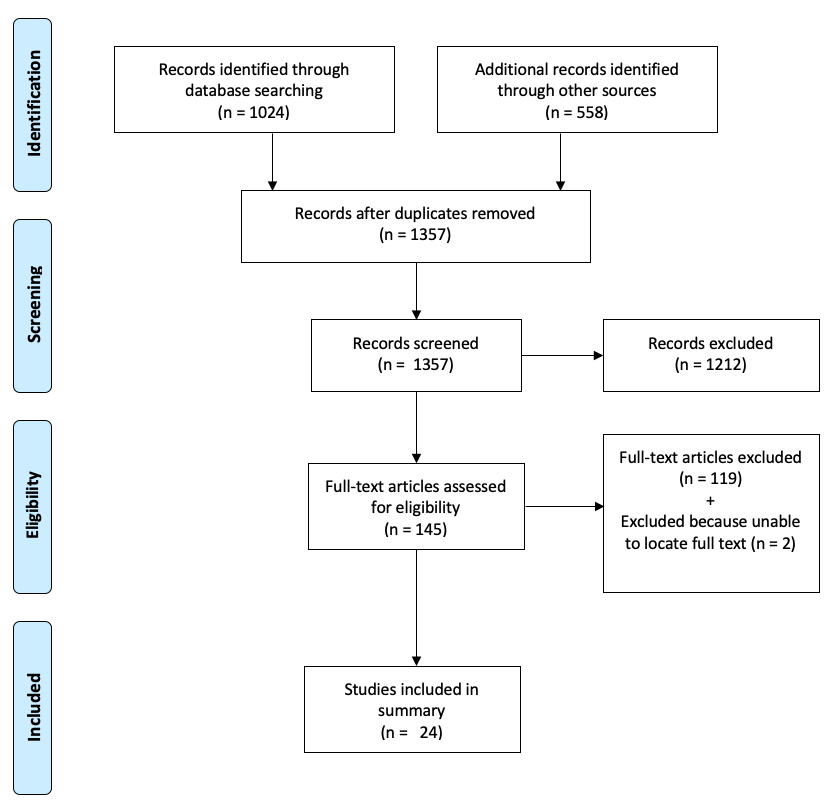

Supplement: Supplementary material [file sj-docx-1-joh-10.1177_00207314211007100.docx]
